# Effectiveness of Teriparatide in Women Over 75 Years of Age with Severe Osteoporosis: 36-Month Results from the European Forsteo Observational Study (EFOS)

**DOI:** 10.1007/s00223-012-9590-9

**Published:** 2012-04-01

**Authors:** J. Bernard Walsh, Willem F. Lems, Dimitrios Karras, Bente L. Langdahl, Osten Ljunggren, Astrid Fahrleitner-Pammer, Annabel Barrett, Gerald Rajzbaum, Franz Jakob, Fernando Marin

**Affiliations:** 1 St. James’s Hospital and Trinity College, Dublin, Ireland; 2 VU University Hospital, Amsterdam, The Netherlands; 3 Veterans Administration Hospital, Athens, Greece; 4 University Hospital, Aarhus, Demark; 5 Department of Medical Sciences, Uppsala University, Uppsala, Sweden; 6 Medical University, Graz, Austria; 7 Lilly Research Centre, Windlesham, UK; 8 St. Joseph Hospital, Paris, France; 9 Julius-Maximillians University, Wuerzburg, Germany; 10 Orthopedic Center for Musculoskeletal Research, Experimental and Clinical Osteology, University of Wuerzburg, Brettreichstrasse 11, 97074 Wuerzburg, Germany

**Keywords:** Age, Back pain, Fracture, Health-related quality of life, Osteoporosis, Teriparatide

## Abstract

This predefined analysis of the European Forsteo Observational Study (EFOS) aimed to describe clinical fracture incidence, back pain, and health-related quality of life (HRQoL) during 18 months of teriparatide treatment and 18 months post-teriparatide in the subgroup of 589 postmenopausal women with osteoporosis aged ≥75 years. Data on clinical fractures, back pain (visual analogue scale, VAS), and HRQoL (EQ-5D) were collected over 36 months. Fracture data were summarized in 6-month intervals and analyzed using logistic regression with repeated measures. A repeated-measures model analyzed changes from baseline in back pain VAS and EQ-VAS. During the 36-month observation period, 87 (14.8 %) women aged ≥75 years sustained a total of 111 new fractures: 37 (33.3 %) vertebral fractures and 74 (66.7 %) nonvertebral fractures. Adjusted odds of fracture was decreased by 80 % in the 30 to <36–month interval compared with the first 6-month interval (*P* < 0.009). Although the older subgroup had higher back pain scores and poorer HRQoL at baseline than the younger subgroup, both age groups showed significant reductions in back pain and improvements in HRQoL postbaseline. In conclusion, women aged ≥75 years with severe postmenopausal osteoporosis treated with teriparatide in normal clinical practice showed a reduced clinical fracture incidence by 30 months compared with baseline. An improvement in HRQoL and, possibly, an early and significant reduction in back pain were also observed, which lasted for at least 18 months after teriparatide discontinuation when patients were taking other osteoporosis medication. The results should be interpreted in the context of an uncontrolled observational study.

Osteoporotic fractures are a significant cause of morbidity and mortality [1–3]. Such fractures, especially vertebral fractures, can cause back pain, can reduce mobility and physical functioning, and may impair health-related quality of life (HRQoL) [4–6]. Age is an independent predictor of osteoporosis and of all types of fracture [7–9]. The risk of fracture is higher in older postmenopausal women than in younger ones, even at the same level of bone mineral density (BMD) [10]. The population of women aged 80 years or over is steadily increasing [11], and it has been estimated that they contribute about 30 % of all fragility fractures and more than 60 % of all nonvertebral fractures [12].

Osteoporosis treatment aims to reduce the risk of fracture, but evidence on the efficacy and use of medications in those at greatest risk, including elderly frail subjects, is limited. Subgroup analyses from pivotal randomized controlled trials (RCTs) have indicated that osteoporosis medications, such as calcium and vitamin D, alendronate, risedronate, zoledronic acid, strontium ranelate, and denosumab, are effective at reducing the fracture risk in older postmenopausal women with osteoporosis [13–20]. Teriparatide (rhPTH1–34) is a bone anabolic agent used to treat postmenopausal women and men with severe osteoporosis who are at high risk of fracture. In a subgroup of elderly women aged ≥75 years, teriparatide significantly reduced the absolute risk for new vertebral fractures by 9.9 % compared with the calcium and vitamin D–supplemented placebo group over a median duration of treatment of 19 months (relative risk reduction 65 %), similar to that seen in postmenopausal women younger than 75 years [21]. RCTs have strict inclusion criteria, and their findings may not be wholly applicable to the broader range of osteoporosis patients seen in everyday clinical practice. Observational studies are conducted in the naturalistic setting without randomization to treatment or exclusion of patients of advanced age and/or those with comorbidities and taking comedications; thus, they may have wider applicability [22].

The European Forsteo Observational Study (EFOS) was an observational study in postmenopausal women with osteoporosis treated with teriparatide in normal clinical practice [23]. The participating women were aged between 39 and 92 years and were treated with teriparatide (20 μg once daily by subcutaneous injection) for up to 18 months and followed by a post-teriparatide treatment period of a further 18 months. The overall fracture outcomes and back pain over the 36-month follow-up for the total study cohort were reported recently [24].

The aim of the present predefined analysis was to describe the fracture outcomes, back pain, and HRQoL of the subgroup of older women aged ≥75 years and to compare the findings with those of the younger participants (<75 years) of EFOS.

## Methods

### Study Design and Patients

EFOS was a multicenter, prospective, observational study conducted in eight European countries (Austria, Denmark, France, Germany, Greece, Ireland, the Netherlands, and Sweden). The study design and characteristics of the patient population have been described in detail elsewhere [23]. Briefly, 1,649 postmenopausal women with a diagnosis of osteoporosis who were about to initiate teriparatide treatment were enrolled. Patients were followed for the duration of their teriparatide treatment, which they could discontinue at any time, and were asked to return for two additional visits after they discontinued teriparatide. Patients were excluded from the study if they were currently being treated with an investigational drug or procedure or had any contraindications as described in the teriparatide label [25]. The observational design meant there were no further restrictions for the selection of patients, and all patient care provided was according to the clinical judgment and usual practice of the participating physicians.

Women provided written informed consent prior to enrollment and were able to withdraw without consequence at any time. The study was approved by local ethics committees or review boards, depending on local requirements, and was conducted in accordance with the ethical standards of the Declaration of Helsinki. The study was conducted from April 2004 (first patient enrolled) until February 2009 (last patient completed).

### Data Collection and Outcomes

Data collected at the baseline visit included patient demographic characteristics, risk factors for osteoporosis and falls, drugs related to the risk of osteoporosis, and disease status [23]. The number and type of prior and current medications for the treatment of osteoporosis were recorded.

Teriparatide (20 μg once daily by self-administered subcutaneous injection) was initiated at the baseline visit, and women attended follow-up visits at approximately 3, 6, 12, and 18 months after teriparatide initiation and at 6 and 18 months after discontinuing teriparatide treatment. All osteoporosis treatment was at the discretion of the physician.

Diagnosis of osteoporosis was based upon axial or peripheral dual-energy X-ray absorptiometric (DXA) measurements of BMD and confirmed following review of medical reports. Incident clinical vertebral and nonvertebral fragility fractures during the observational period were diagnosed and confirmed by review of the original X-rays and/or the radiology or surgical reports at the study site. A new or worsened clinical vertebral fracture was defined as the presence of a confirmed radiographic vertebral fracture associated with signs and/or symptoms suggestive of a new vertebral fracture [26].

Back pain was self-assessed by patients at each study visit using a 100-mm visual analogue scale (VAS), ranging from 0 = no back pain to 100 = worst possible back pain. Patients also completed a back pain questionnaire that captured the frequency and severity of back pain, limitations of activities, and days in bed due to back pain in the previous month [24].

HRQoL was measured at each visit using the European Quality of Life Questionnaire (EQ-5D) [27], where patients assess their perceived overall health status on a visual analogue scale (EQ-VAS) that ranges from 0 = worst imaginable health state to 100 = best imaginable health state and classify their own health status according to five dimensions of health (mobility, self-care, usual activities, pain/discomfort, and anxiety/depression), each of which is scored on a three-point scale (no problems, some problems, or extreme problems). The UK scoring algorithm was used to calculate a single summary index, the Health State Value (HSV), from the five EQ-5D dimensions [28].

### Statistical Analysis

For data analyses, the total study cohort included all patients with a baseline visit and at least one follow-up visit. Patients were retrospectively categorized into one of two subgroups according to their age at baseline: ≥75 or <75 years.

Descriptive statistics, such as frequencies, percentages, means, standard deviations (SDs), and ranges, were used to describe patients in the two subgroups. Between-group comparisons were made using chi-squared or Fisher’s exact tests (categorical variables) or the Kruskal-Wallis test (continuous variables).

The number of fractures occurring in patients aged ≥75 or <75 years was summarized in 6-month intervals. For each subgroup, a logistic regression with repeated measures was used to assess the change in number of patients with one or more fractures over time [29, 30], giving an analysis of the odds of one or more fractures, as described for the total study cohort [24]. Patients were included in the model at all observed intervals, irrespective of whether or not they had fractured during a previous interval. The repeated observations of each patient were assumed to be related, but no further assumptions were made about the relationship. Unadjusted and adjusted analyses (including age, prior bisphosphonate use, and fracture in the last 12 months before starting teriparatide) were performed. Contrasts were made between the odds of fracture in the first 6 months of treatment (0 to <6 months) and each subsequent 6-month interval.

Back pain and HRQoL were summarized over the teriparatide treatment period and after teriparatide discontinuation for both age groups. Changes in back pain VAS from baseline were analyzed using a mixed model of repeated measures (MMRM), adjusting for back pain VAS at baseline, number of previous fractures, age, diagnosis of rheumatoid arthritis, duration of prior bisphosphonate therapy, and history of fracture in the 12 months before entering the study. Changes from baseline in severity of back pain, frequency of back pain, and limitation of activities due to back pain at each follow-up visit were categorized as improvement, no change, or worsening (defined by a change in the categorical response for each question in the back pain questionnaire); and the number/percent of patients improving/worsening was analyzed using the sign test.

A similar MMRM was used to assess the change from baseline in EQ-VAS, including its baseline value. The sign test analyzed the number/percent of patients reporting an improvement or worsening from baseline in each of the five EQ-5D domains, which was determined by assigning a number to each of the three possible responses for each domain and then determining whether the numerical difference between baseline and each time point was an increase (improvement), no change, or a decrease (worsening). Changes from baseline in EQ-5D HSV were assessed using the Wilcoxon sign-rank test because this parameter has a nonparametric distribution.

## Results

**Fig. 1:**
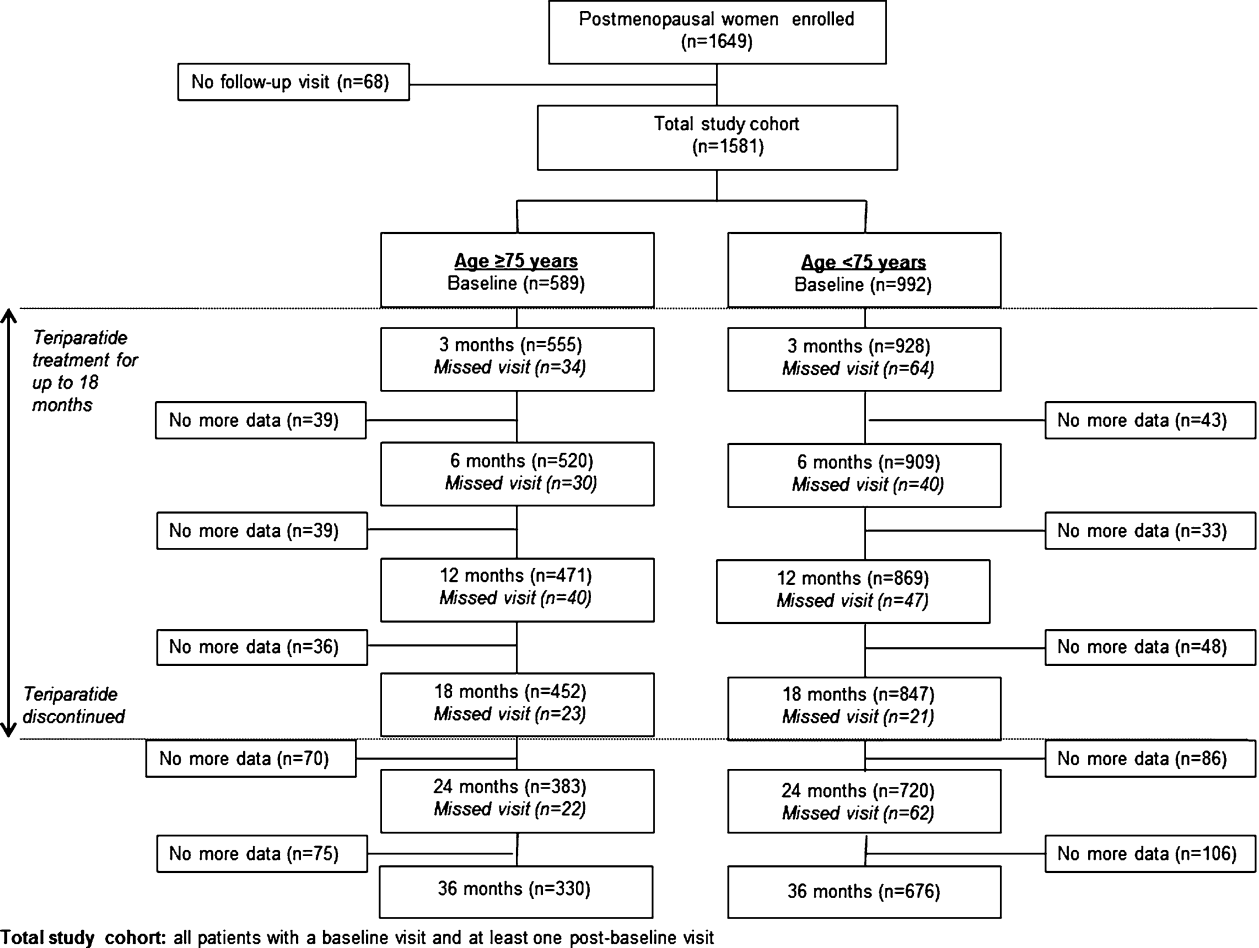
Patient disposition stratified by age <75 and ≥75 years

Of the 1,581 patients in the total study cohort, 589 (37 %) were aged ≥75 years and 992 (63 %) were <75 years. The disposition of patients during the study by age subgroup (Fig. 1) shows that 76.7 and 56.0 % of patients aged ≥75 years completed the 18- and 36-month visits, respectively.

Table 1 summarizes the demographic and baseline characteristics of the patients in the two age groups (≥75, <75 years). At baseline, the older and younger subgroups had similar lumbar spine BMD, prior bisphosphonate use, and time since most recent fracture. However, the older subgroup reported a higher frequency of risk factors associated with falls and fractures (such as sight problems), a higher number of prevalent fractures, and a history of falls. Significantly more patients in the elderly subgroup had diabetes mellitus or dementia and were taking concomitant medications including antihypertensives, benzodiazepines, antiarrhythmics, and anticoagulants/heparin. Furthermore, the older subgroup had a higher mean back pain VAS and poorer HRQoL as measured by the EQ-5D at baseline compared with patients aged <75 years (Table 1).

**Table 1:** Baseline characteristics of the total study cohort (*n* = 1,581) stratified by age

Characteristic	Age <75 years	Age ≥75 years	*P*
Patients, *n* (%)	992 (62.7)	589 (37.2)	
Mean age, years (SD; range)	66.1 (6.4; 39–74)	79.2 (3.6; 75–92)	NA
Caucasian (%)	99.1	99.4	0.550
Mean body mass index (SD)	25.3 (4.3)	24.9 (4.3)	0.173
Early menopause (<40 years) (%)	11.0	5.3	**<0.001**
Surgical menopause (%)	20.6	15.5	**0.013**
Nulliparous (%)	13.4	12.3	0.540
Sight problems (%)	37.5	57.7	**<0.001**
Osteoporotic hip fracture in biological mother (%)	22.6	17.7	**0.036**
Current smoker (%)	17.5	5.5	**<0.001**
Mean lumbar spine BMD T score (SD)	−3.3 (1.1)	−3.2 (1.3)	0.442
Bisphosphonate prior use (%)	72.2	75.6	0.142
Previous fracture (%)	85.8	92.0	**<0.001**
Previous fractures after 40 years of age, mean (SD)	2.7 (1.9)	3.3 (2.0)	**<0.001**
≥4 fractures after 40 years of age (%)	31.6	42.9	**<0.001**
Median time since most recent fracture, years (IQR)	0.7 (0.2–2.4)	0.7 (0.0–2.4)	0.888
At least one fracture in 12 months prior to study (%)	47.1	50.6	0.176
Assist with arms when standing up from chair (%)	59.9	69.1	**<0.001**
>1 fall in the last year (%)	21.0	26.5	**0.009**
Back pain and HRQoL			
Mean back pain VAS, mm (SD)	56.5 (26.8)	59.8 (26.1)	**0.019**
Mean EQ-VAS (SD)	53.6 (21.8)	49.3 (22.0)	**<0.001**
Median EQ-HSV (IQR)	0.62 (0.09–0.73)	0.52 (0.00–0.69)	**<0.001**
Comorbidities (%)^a^			
Rheumatoid arthritis	12.6	10.7	0.258
Chronic obstructive pulmonary disease	9.4	7.6	0.237
Diabetes mellitus	4.4	7.3	**0.016**
Dementia	0.1	1.0	**0.008**
Concomitant medication (taken at study entry) (%)^a^	61.3	68.0	**0.008**
Antihypertensives	34.4	42.0	**0.003**
Glucocorticoids	15.1	14.4	0.717
Benzodiazepines	10.6	14.4	**0.025**
Thyroid hormones	14.0	12.2	0.290
Antiarrhythmics	5.5	11.6	**<0.001**
Anticoagulants/heparin	4.8	10.3	**<0.001**

*P* values for group differences were calculated using *t* tests, Kruskal-Wallis tests, chi-squared tests, or Fisher’s exact test

^a^The three most frequent are listed plus any others that were significantly different between groups

*SD* standard deviation, *BMD* bone mineral density, *IQR* interquartile range, *HRQoL* health-related quality of life, *EQ-VAS* European Quality of Life Questionnaire (EQ-5D) Visual Analogue Scale, *EQ-HSV* European Quality of Life Questionnaire (EQ-5D) Health State Value

The statistically significant *P* values in bold

### Treatment

Median duration of teriparatide treatment was similar in the two age groups: 543 days (Q1, Q3: 478, 553) for patients ≥75 years and 543 days (Q1, Q3: 525, 555) for patients <75 years. The reasons for discontinuation of teriparatide in the subgroups aged ≥75 and <75 years, respectively, were treatment completed (70.4 and 81.9 %), patient decision (13.9 and 9.0 %), adverse event (9.0 and 6.8 %), physician decision (4.4 and 1.8 %), death (1.5 and 0.8 %), and noncompliance (0.8 and 0.3 %).

Of the 298 patients aged ≥75 years with data available on osteoporosis medication taken after teriparatide was discontinued, 95.6 % took some type of osteoporosis medication. Most women took calcium (83.6 %) and vitamin D (85.6 %), and 63.4 % were prescribed a bisphosphonate. Likewise, of the 611 patients aged <75 years with data on osteoporosis medication taken after teriparatide was discontinued, 93.6 % took an osteoporosis medication, mainly a bisphosphonate (63.2 %), with most women also taking calcium (84.9 %) and vitamin D supplementation (86.5 %).

### Fracture Outcomes

Table 2 summarizes the fracture incidence during teriparatide treatment (0 to <18 months) and after teriparatide discontinuation (18 to <36 months) in the older (≥75 years) and younger (<75 years) subgroups. In the older subgroup, 87 (14.8 %) women sustained one or more fractures during the 36-month follow-up: 68 women sustained a single fracture and 19 sustained two or more fractures. Of the 111 fractures, 37 (33.3 %) were clinical vertebral fractures and 74 (66.7 %) were nonvertebral fractures; of all fractures, 54 (48.6 %) were main-site nonvertebral fractures: forearm/wrist (*n* = 16), leg (*n* = 13), hip (*n* = 11), sternum/ribs (*n* = 8), and humerus (*n* = 6). The adjusted odds of fractures was not reduced during the teriparatide treatment phase in the older subgroup but was significantly lower in the last two time intervals (24 to <30 months and 30 to <36 months) compared with the first 6-month interval (0 to <6 months); there was an 80 % decrease in the odds of fracture in the 30 to <36–month period compared to the first 6-month period (*P* < 0.009) (Table 2).

**Table 2:** Fracture incidence during teriparatide treatment (0 to <18 months) and after teriparatide was discontinued (18 to <36 months) in patients aged ≥75 and <75 years

Time interval (months)	*n* (missing/ unknown)	Fractures/ 10,000 patient years	Total fractures	Patients with ≥1 fracture, *n* (%)^a^	OR^b,c^ (95 % CI)	*P*
Age ≥75 years						
0 to <6	586 (3)	1,022	29	26 (4.4)	–	–
6 to <12	537 (1)	1,292*	33	28 (5.2)	1.17 (0.68–2.03)	0.564
12 to <18	485 (0)	978**	23	23 (4.7)	1.07 (0.60–1.90)	0.824
18 to <24	443 (1)	636	13	13 (2.9)	0.65 (0.33–1.27)	0.203
24 to <30	369 (3)	474	8	7 (1.9)	0.42 (0.18–0.98)	**0.044**
30 to <36	324 (0)	333	5	3 (0.9)	0.20 (0.06–0.67)	**0.009**
Total	586 (3)		111	87 (14.8)		
Age <75 years						
0 to <6	990 (2)	1,176	57	50 (5.0)	–	–
6 to <12	936 (1)	548	25	23 (2.5)	0.47 (0.30–0.76)	**0.002**
12 to <18	885 (1)	464	20	18 (2.0)	0.39 (0.22–0.67)	**0.001**
18 to <24	826 (1)	590	23	21 (2.5)	0.49 (0.29–0.84)	**0.009**
24 to <30	736 (1)	345	12	11 (1.5)	0.29 (0.15–0.56)	**<0.001**
30 to <36	667 (0)	324	10	10 (1.5)	0.29 (0.15–0.57)	**<0.001**
Total	990 (2)		147	121 (12.2)		

*n* is the number of patients who attended the observation (number of patients with fracture data missing or unknown at this observation)

^a^As some patients experienced a fracture in more than one time interval, the total was not the sum of patients with a fracture in each interval

^b^Adjusted model by age, prior bisphosphonate use, and fracture in past 12 months before starting teriparatide

^c^Compared with 0 to <6 month interval

* *P* = 0.010 compared with the younger group, ** *P* = 0.008 compared with the younger group

The statistically significant *P* values in bold

In the subgroup of women aged <75 years, 121 (12.2 %) sustained a total of 147 clinical fractures during the 36-month follow-up (Table 2): 102 women (84.3 %) had a single fracture and 19 (15.7 %) had two or more fractures. Of the 147 fractures, 50 (34.0 %) were clinical vertebral fractures and 97 (66.0 %) were nonvertebral fractures, including 25, 16, 15, 11 and 8 fractures at the forearm/wrist, hip, humerus, sternum/ribs, and leg, respectively. There was a 71 % decrease in the odds of fracture in the 30 to <36–month period compared with the first 6-month period (*P* < 0.001) (Table 2).

Comparisons between the two age groups at each time period showed that patients aged ≥75 years had a significantly higher adjusted odds of fracture at 6 to <12 months (OR = 2.11, 95 % CI 1.20–3.70; *P* = 0.010) and at 12 to <18 months (OR = 2.33, 95 % CI 1.24–4.35; *P* = 0.008). There was no difference in the odds of fracture between the older and younger groups at the other time intervals.

### Back Pain

**Fig. 2:**
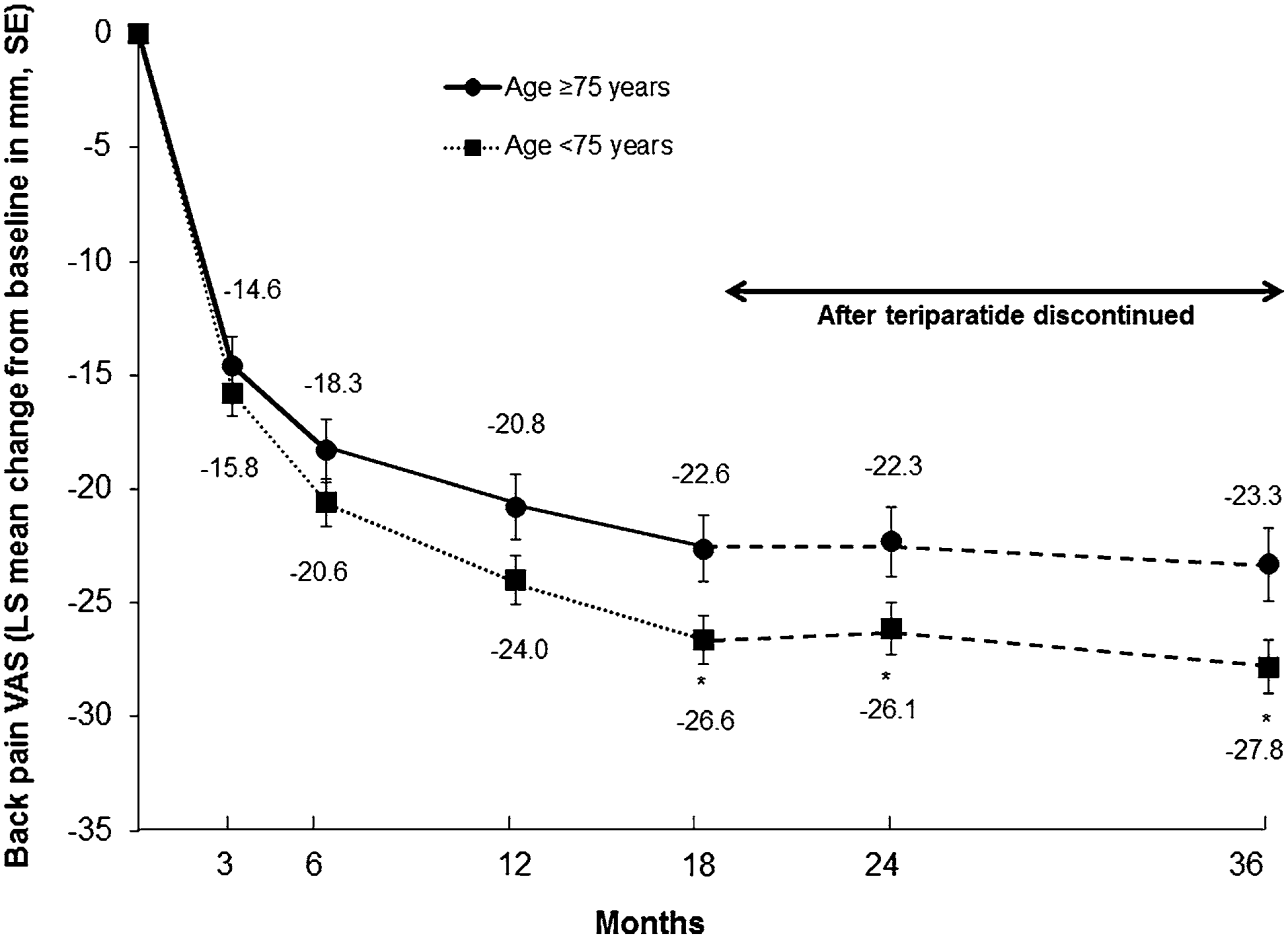
Back pain VAS: least squares (LS) mean change from baseline during and after teriparatide treatment in subgroups of patients aged <75 and ≥75 years. Back pain VAS range 0–100 mm. All values in both groups *P* < 0.001 versus baseline. *P < 0.05 versus elderly subgroup (age ≥75 years). Data are from MMRM analysis. Model included baseline back pain VAS, number of previous fractures, fracture in 12 months before study entry, age, prior bisphosphonate duration, diagnosis of rheumatoid arthritis, and visit, where repeated measures were modeled with an unstructured correlation matrix. In the elderly subgroup (≥75 years), the unadjusted mean (SD) back pain VAS scores at baseline, 3, 6, 12, 18, 24, and 36 months and end of study (LOCF) were 59.8 (26.1), 44.0 (24.1), 39.5 (23.9), 36.8 (24.3), 34.7 (24.3), 34.9 (26.2), 31.4 (26.1) and 37.3 (27.1) mm, respectively. The corresponding values for the younger subgroup (<75 years) were 56.5 (26.8), 42.3 (25.5), 37.7 (26.2), 33.4 (26.3), 30.4 (26.0), 30.7 (26.9), 28.3 (26.4), and 31.3 (27.2) mm

The older subgroup of patients (≥75 years) had significantly higher mean back pain VAS scores (unadjusted) at baseline and at 12, 18, and 24 months compared with the younger subgroup (<75 years). There were statistically significant reductions in adjusted back pain VAS scores from baseline in both subgroups at each postbaseline visit (Fig. 2). The decrease in back pain seen during teriparatide treatment was maintained during the 18 months after teriparatide discontinuation. The reduction in back pain was slightly greater in the younger group of patients; in the adjusted model, the difference between age groups was significant at 18, 24, and 36 months (*P* < 0.05) but was less than 5 mm at each visit (i.e., of little clinical significance). Of the variables included in the MMRM, three were significantly associated with the change in back pain VAS: each additional 5 mm in baseline back pain VAS was associated with a greater reduction in back pain of −2.89 mm (95 % CI −2.71 to −3.07; *P* < 0.001), a fracture in the 12 months before study entry was associated with a greater reduction in back pain VAS of −2.48 mm (95 % CI −0.55 to −4.42; *P* = 0.0118) vs. no fracture, and each additional previous fracture was associated with an increase in back pain VAS of 1.09 mm (95 % CI 0.61–1.58; *P* < 0.001).

Table 3 summarizes the results from the back pain questionnaire on back pain frequency, severity, and limitations of activities due to back pain both during and after teriparatide treatment for patients ≥75 and <75 years. Compared with the younger group, the older patients had a higher frequency of back pain at baseline and at 3 and 6 months and a higher severity of back pain at baseline and 3 months. The older subgroup also had more severe limitations of activities at every time point.

**Table 3:** Back pain questionnaire results for subgroups of patients ≥75 and <75 years

		During teriparatide treatment period				After teriparatide discontinued		
	Baseline	3 months	6 months	12 months	18 months	24 months	36 months	End of study (LOCF)^f^
Frequency of back pain^a^								
Age ≥75 years	(*n* = 584)	(*n* = 544)	(*n* = 513)	(*n* *=* 463)	(*n* *=* 444)	(*n* *=* 376)	(*n* *=* 320)	(*n* *=* 557)
Every day/almost every day (%)	68.8*	39.3*	34.3*	32.2	28.8	27.7	22.5	31.4
Age <75 years	(*n* *=* 985)	(*n* *=* 916)	(*n* *=* 898)	(*n* *=* 858)	(*n* *=* 827)	(*n* *=* 708)	(*n* *=* 670)	(*n* *=* 962)
Every day/almost every day (%)	59.8	33.2	31.3	27.0	23.9	23.0	20.3	24.2
Severity of back pain^b^								
Age ≥75 years	(*n* *=* 551)	(*n* *=* 503)	(*n* *=* 465)	(*n* *=* 417)	(*n* *=* 388)	(*n* *=* 315)	(*n* *=* 262)	(*n* *=* 475)
Severe (%)	51.0*	20.9*	14.8	14.4	11.6	13.0	11.5	16.0
Age <75 years	(*n* *=* 930)	(*n* *=* 817)	(*n* *=* 775)	(*n* *=* 675)	(*n* *=* 640)	(*n* *=* 532)	(*n* *=* 486)	(*n* *=* 730)
Severe (%)	41.9	17.0	14.6	13.2	11.4	13.0	12.6	15.3
Limitation of activities due to back pain^c^								
Age ≥75 years	(*n* *=* 552)	(*n* *=* 504)	(*n* *=* 465)	(*n* *=* 417)	(*n* *=* 390)	(*n* *=* 316)	(*n* *=* 262)	(*n* *=* 475)
Severe (%)	42.6*	18.5*	11.8*	14.6*	13.1*	15.2*	13.7*	17.1*
Age <75 years	(*n* *=* 930)	(*n* *=* 815)	(*n* *=* 773)	(*n* *=* 675)	(*n* *=* 641)	(*n* *=* 536)	(*n* *=* 487)	(*n* *=* 731)
Severe (%)	34.2	16.2	13.6	10.2	8.6	11.4	11.3	13.4
Days in bed due to back pain^d^								
Age ≥75 years	(*n* *=* 549)	(*n* *=* 502)	(*n* *=* 467)	(*n* *=* 415)	(*n* *=* 388)	(*n* *=* 314)	(*n* *=* 261)	(*n* *=* 473)
At least 1 (%)	23.5	9.8	9.4	8.4	8.0	10.5	8.8	10.6
Median (Q1, Q3)^e^	10 (3, 20)	5 (3, 10)	3 (2, 8)	4 (2, 10)	7 (2, 11)	4 (2, 10)	3 (2, 6)	4 (2, 10)
Age <75 years	(*n* *=* 930)	(*n* *=* 816)	(*n* *=* 773)	(*n* *=* 675)	(*n* *=* 640)	(*n* *=* 536)	(*n* *=* 486)	(*n* *=* 732)
At least 1 (%)	19.8	7.4	5.0	4.4	3.9	6.0	4.3	6.4
Median (Q1, Q3)^e^	6 (3, 15)	3 (2, 7)	3 (1, 5)	3 (2, 5)	3 (2, 6)	3 (2, 7)	3 (2, 4)	3 (2, 6)

Total *n* varies for each variable due to missing data. The percentages given for each variable refer to the total *n* available for that variable

^a^Categories were no pain, once or twice, a few times, fairly often, every day or almost every day (during the past month)

^b^Categories were minor, moderate, severe (during the past month)

^c^Categories were no limitation, minor extent, moderate extent, severe extent (during the past month)

^d^In the past month

^e^For those patients with at least 1 day in bed due to back pain during the last month

^f^Missing data were handled using the last observation carried forward (LOCF) method

* *P* < 0.05 for comparison with patients aged <75 years (Cochran-Mantel-Haenzsel test). A greater percentage of patients in both age groups reported an improvement than a worsening relative to baseline at all postbaseline visits (sign test, *P* < 0.001)

At every postbaseline visit, more patients in both subgroups reported an improvement than a worsening in back pain frequency relative to baseline (sign test, *P* < 0.001). The same was true for severity of back pain and limitation in activities due to back pain.

### Health-Related Quality of Life

**Fig. 3:**
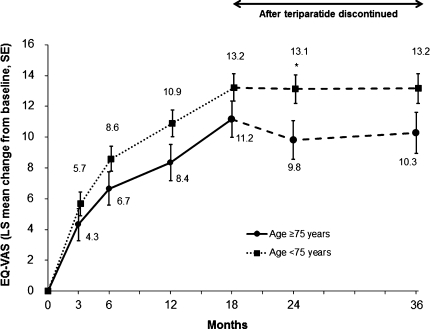
HRQoL: least squares (LS) mean change from baseline in EQ-VAS in older (≥75 years) and younger (<75 years) women both during teriparatide treatment for 18 months and in the 18 months after teriparatide was discontinued. EQ-VAS range 0–100. All values in both groups *P* < 0.001 versus baseline. *P < 0.001 versus elderly subgroup (age ≥75 years). Model includes baseline EQ-VAS score, number of previous fractures, fracture in 12 months before study entry, age, prior bisphosphonate use duration, and diagnosis of rheumatoid arthritis. For women aged ≥75 years, unadjusted mean (SD) EQ-VAS values at baseline, 3, 6, 12, 18, 24, and 36 months and end of study (LOCF) were 49.3 (22.0), 55.7 (19.2), 58.6 (19.2), 60.5 (20.9), 63.8 (20.9), 63.2 (22.2), 65.4 (22.2), and 60.4 (22.7), respectively. The corresponding values for the younger women were 53.6 (21.8), 60.9 (20.0), 63.8 (20.3), 66.7 (21.4), 69.4 (21.4), 69.6 (22.3), 70.3 (22.4), and 67.4 (23.2)

There were significant improvements from baseline in adjusted mean EQ-VAS at all postbaseline visits in both age groups of patients (Fig. 3). The increase in adjusted EQ-VAS score was significantly higher in the younger age group compared with the older age group at the 24-month visit only. The unadjusted mean EQ-VAS scores show that the older patients (≥75 years) had a significantly poorer HRQoL at baseline and at all follow-up visits (all *P* < 0.05) compared with the younger patients (<75 years).

A significantly higher percentage of patients in the older subgroup reported some/extreme problems for each of the EQ-5D domains compared with the younger age group (Table 4). For all five domains at all postbaseline visits, a greater percentage of patients in both age groups reported an improvement than a worsening relative to baseline (sign test, *P* < 0.001).

**Table 4:** Percentage of patients aged ≥75 and <75 years reporting problems (some/extreme) in each of the EQ-5D domains

		During teriparatide treatment				After teriparatide discontinued		
	Baseline	3 months	6 months	12 months	18 months	24 months	36 months	End of study (LOCF)^a^
Mobility								
≥75 years	(*n* *=* 578)	(*n* *=* 527)	(*n* *=* 503)	(*n* *=* 455)	(*n* *=* 426)	(*n* *=* 368)	(*n* *=* 301)	(*n* *=* 516)
	79.9**	67.6**	61.4**	57.8**	58.2**	56.0**	56.1**	61.8**
<75 years	(*n* *=* 989)	(*n* *=* 898)	(*n* *=* 886)	(*n* *=* 843)	(*n* *=* 813)	(*n* *=* 695)	(*n* *=* 640)	(*n* *=* 904)
	63.1	48.7	44.0	40.1	36.5	35.7	34.5	38.7
Self-care								
≥75 years	(*n* *=* 578)	(*n* *=* 527)	(*n* *=* 501)	(*n* *=* 454)	(*n* *=* 427)	(*n* *=* 368)	(*n* *=* 301)	(*n* *=* 517)
	53.3**	42.9**	36.3**	34.8**	34.4**	34.2**	36.5**	40.2**
<75 years	(*n* *=* 984)	(*n* *=* 901)	(*n* *=* 886)	(*n* *=* 843)	(*n* *=* 814)	(*n* *=* 695)	(*n* *=* 642)	(*n* *=* 906)
	37.5	24.4	22.3	19.7	19.3	17.4	19.2	21.6
Usual activities								
≥75 years	(*n* *=* 576)	(*n* *=* 523)	(*n* *=* 501)	(*n* *=* 455)	(*n* *=* 426)	(*n* *=* 368)	(*n* *=* 300)	(*n* *=* 516)
	81.6**	74.4**	68.3**	68.4**	62.4**	63.0**	62.3**	68.4**
<75 years	(*n* *=* 987)	(*n* *=* 897)	(*n* *=* 884)	(*n* *=* 840)	(*n* *=* 814)	(*n* *=* 693)	(*n* *=* 641)	(*n* *=* 904)
	73.8	59.5	51.2	48.3	45.0	41.8	43.8	48.6
Pain and discomfort								
≥75 years	(*n* *=* 575)	(*n* *=* 523)	(*n* *=* 496)	(*n* *=* 454)	(*n* *=* 425)	(*n* *=* 366)	(*n* *=* 301)	(*n* *=* 513)
	93.9**	90.2**	85.7**	84.6**	80.7**	79.2**	72.1**	80.5**
<75 years	(*n* *=* 979)	(*n* *=* 900)	(*n* *=* 881)	(*n* *=* 841)	(*n* *=* 812)	(*n* *=* 696)	(*n* *=* 642)	(*n* *=* 906)
	91.5	81.8	75.9	72.4	67.2	64.4	62.0	67.8
Anxiety and depression^b^								
≥75 years	(*n* *=* 578)	(*n* *=* 525)	(*n* *=* 502)	(*n* *=* 454)	(*n* *=* 426)	(*n* *=* 368)	(*n* *=* 301)	(*n* *=* 516)
	62.3*	53.1*	49.4*	47.8*	44.6*	49.7**	43.7*	49.4**
<75 years	(*n* *=* 986)	(*n* *=* 900)	(*n* *=* 884)	(*n* *=* 843)	(*n* *=* 814)	(*n* *=* 695)	(*n* *=* 641)	(*n* *=* 905)
	54.9	46.9	42.1	41.2	39.2	39.7	36.4	39.9

The *n* varies for each variable and at each time point due to missing data. The percentage given for each variable refers to the total *n* available for that variable

^a^Missing data were handled using the last observation carried forward (LOCF) method

^b^Percentages of patients who reported being moderately/extremely anxious or depressed

* *P* < 0.05, ** *P* < 0.001 for the comparison with patients <75 years (Cochran-Mantel-Haenzsel test). A greater percentage of patients in both age groups reported an improvement than a worsening relative to baseline at all postbaseline visits (sign test, *P* < 0.001)

In the older subgroup of women, median (Q1, Q3) HSVs were increased significantly (Wilcoxon signed-rank test, *P* < 0.001) from baseline at all postbaseline visits and were 0.69 (0.52, 0.80) at both 18 and 36 months. The same was observed in the younger age group, where the median (Q1, Q3) HSVs were 0.73 (0.62, 1.00) and 0.76 (0.62, 1.00) at 18 and 36 months, respectively.

## Discussion

The results of this observational study show that elderly women aged 75 years or above with severe postmenopausal osteoporosis treated with teriparatide for up to 18 months had a reduced clinical fracture incidence by 30 and 36 months compared with the first 6 months of treatment. In addition, these older patients reported an early and significant reduction in back pain and improvement in HRQoL during teriparatide treatment, which was sustained after teriparatide was discontinued, during which time most patients were receiving some osteoporosis medication, mainly calcium, vitamin D, and a bisphosphonate. Of note, the majority of the elderly patients who received teriparatide had previously been treated with a potent antiresorptive for their severe bone disease and were also receiving calcium and vitamin D supplements during the course of teriparatide treatment. These findings suggest that teriparatide is an effective treatment in elderly postmenopausal women aged 75 years and above at very high risk of fracture when used as part of a sequential treatment regimen.

Age is a major determinant of bone strength and fracture risk in postmenopausal women. Bone loss (both trabecular and cortical) occurs with advancing age, with decreases in both bone mass and bone quality [31]. Bone fragility increases with age because of changes in microarchitecture and an accumulation of microdamage and hypomineralization [12]. In elderly women with postmenopausal osteoporosis, age-related bone loss is superimposed on loss of bone caused by estrogen deficiency [31, 32]. Moreover, vitamin D deficiency is common in elderly people and can lead to secondary hyperparathyroidism and PTH-induced bone loss [31].

Our findings in patients treated in normal life conditions are consistent with and complement those of the randomized, placebo-controlled Fracture Prevention Trial, where the incidence of new vertebral fractures during approximately 18 months of teriparatide treatment (relative to placebo) was comparable in younger (<75 years) and older (≥75 years) patients [21]. Thus, in women aged 75 and above, 5.2 % in the teriparatide group and 15.1 % in the placebo group had new vertebral fractures (relative risk reduction 65 %).

Because this is an observational, nonrandomized study, it is difficult to compare the incidence of fractures between the two age groups. Nevertheless, the adjusted odds of fracture were higher in the elderly group during the 6–18 months of teriparatide treatment compared with the younger group but were similar between age groups during the 18-month post-teriparatide treatment period. The higher fracture risk during the 6–18 months of teriparatide treatment in patients aged ≥75 years may be a consequence of the additional age-related risk factors in these older women and their higher risk of fracture at baseline, as reflected in the greater frequency of prevalent fractures, falls, and dementia as well as use of concomitant medications.

Evidence for the antifracture efficacy of other antiosteoporosis drugs in postmenopausal women aged ≥75 years with osteoporosis is also based on subgroup analyses of data from randomized, placebo-controlled studies. These studies have shown that treatment with alendronate, risedronate, zoledronic acid, strontium ranelate, and denosumab is associated with a reduction in the risk of new clinical fractures after 3 years [14–18, 20, 33]. Data from the Fracture Intervention Trial showed that alendronate reduced the risk of new vertebral fractures by 38 % (relative to placebo) in the subgroup of women aged ≥75 years during an average follow-up of 2.9 years [14] and that the absolute risk reduction for combined clinical hip, spine, and wrist fractures was greatest in the 75–85 year age group [33]. For risedronate, there was a significant 44 % reduction in the risk of vertebral fractures (relative to placebo) in women aged 80 years or above but no significant difference in the incidence of nonvertebral fractures [15]. A pooled analysis of RCTs in postmenopausal women with osteoporosis found that, despite a 4 % increase in the risk of fracture for every 1-year increase in age, there was a 46 % reduction in the risk of clinical fracture over 3 years with risedronate treatment [17]. For zoledronic acid, post hoc subgroup analysis of pooled data from the HORIZON trials found significant reductions (vs. placebo) in the risk of any clinical fracture (35 %), clinical vertebral fractures (66 %), and nonvertebral fractures (27 %) in postmenopausal women aged ≥75 years [16]. For strontium ranelate, pooled data from the SOTI and TROPOS studies revealed risk reductions of 32 %, 31 %, and 22 % for vertebral, nonvertebral, and clinical fractures after 3 years in women aged 80 years or above with osteoporosis [18]. In a post hoc analysis of the 3-year randomized, double-blind, placebo-controlled FREEDOM study, denosumab treatment significantly reduced the risk of hip fractures by 62 % (relative to placebo) in the subgroup of postmenopausal women aged 75 years or above [20]. Moreover, a recent meta-analysis of the efficacy of antiresorptive therapy in elderly women with osteoporosis found that vertebral fracture risk reduction increased with age and duration of treatment [34].

A main finding of the present study is that both age groups experienced rapid and significant improvements in back pain and HRQoL during teriparatide treatment. These benefits were maintained after teriparatide was discontinued when most patients were receiving other osteoporosis medication. The higher back pain scores and poorer HRQoL in the older subgroup (≥75 years) throughout the study may reflect greater severity of osteoporosis in this subgroup of patients.

There are several limitations of the EFOS study that should be considered when interpreting the data from these analyses. First, as this was an observational study, patients were not randomized by age or to treatment, and there was no comparator group to teriparatide. As a result, we cannot attribute the observed changes to teriparatide treatment. Moreover, as the majority (>93 %) of patients took an osteoporosis medication during the 18-month post-teriparatide treatment period, we cannot exclude the effects of this sequential therapy on the observed responses. Second, we may have underestimated the fracture incidence because only symptomatic vertebral fractures were confirmed by radiology. Third, data on analgesic use during the study were not gathered; this may have affected the back pain results.

The strengths of this study include the large sample size, with no age limit restrictions, and inclusion of a diverse range of patients, many of whom had comorbidities and were taking concomitant medications. Another advantage of the study is that the participating postmenopausal women with severe osteoporosis received teriparatide as part of different sequential therapies, reflecting treatment as it occurs in the normal clinical practice setting. Moreover, we examined fracture incidence, back pain, and quality of life both during and after teriparatide treatment and adjusted for factors that may influence the changes, including previous bisphosphonate use and fracture in the 12 months before starting teriparatide.

The need for osteoporosis medications that are safe and well tolerated is especially important in elderly patients, who are likely to have comorbidities and to be taking concomitant medications. The safety of teriparatide has already been established, and results from the placebo-controlled Fracture Prevention Trial have shown that teriparatide was well tolerated in elderly patients, with no significant differences in the safety profile between patients ≥75 and <75 years [21]. Safety was not an objective of the present observational study.

## Conclusion

Patients with severe postmenopausal osteoporosis aged 75 years or above treated with teriparatide showed a reduced incidence of clinical fractures by 30 months compared with baseline, together with improvements in HRQoL and possibly an early and significant reduction in back pain. These outcomes lasted for at least 18 months after teriparatide discontinuation when patients were taking other osteoporosis medication. These results should be interpreted in the context of an uncontrolled observational study.
